# Prognostic Value of Admission Lactate Levels in Critically Ill Patients: A Comparative Study With Sequential Organ Failure Assessment and Acute Physiology and Chronic Health Evaluation II Scores

**DOI:** 10.7759/cureus.71372

**Published:** 2024-10-13

**Authors:** Abhijeet Kumrawat, Sunita Gupta, Harmanjeet S Dhillon, Purva Kumrawat

**Affiliations:** 1 General Medicine, Maharishi Markandeshwar Institute of Medical Sciences and Research, Mullana, IND; 2 Anaesthesia, Uttar Pradesh University of Medical Sciences, Saifai, IND

**Keywords:** apache ii score, critical care, lactate, medical icu, prognostic markers, sofa

## Abstract

Introduction

Critical illness refers to life-threatening conditions requiring mechanical or pharmacological intervention to maintain organ function. Prognostic models, such as the Sequential Organ Failure Assessment (SOFA) and Acute Physiology and Chronic Health Evaluation (APACHE) II scores, have been widely used to predict mortality in intensive care unit (ICU) patients. Lactate levels are emerging as a valuable biomarker in this context. This study aims to determine the prognostic value of lactate levels upon admission in critically ill patients and to assess their correlation with SOFA and APACHE II scores.

Methods

This descriptive cross-sectional study included 200 critically ill patients admitted to the emergency department over one year. Data on patient demographics, clinical findings, and laboratory results were collected, and lactate levels and SOFA and APACHE II scores were measured at the time of admission. Patients were followed throughout their hospital stay, with outcomes classified as survival or mortality. Receiver operating characteristic (ROC) curve analysis was used to evaluate the predictive value of lactate, SOFA, and APACHE II scores. Statistical analysis was performed using IBM SPSS Statistics for Windows, Version 27.0 (Released 2020; IBM Corp., Armonk, New York, United States).

Results

The mean age of the patients was 56.8±16.9 years; 110 (55%) were men, and 90 (45%) were women. In total, 79 patients (39.5%) were non-survivors, and 121 (60.5%) were survivors. Lactate levels were significantly higher in non-survivors (3.56±1.90 mmol/L) compared to survivors (1.47±0.82 mmol/L) (p<0.001). The SOFA and APACHE II scores were also significantly higher in non-survivors (SOFA: 6.35±3.19; APACHE II: 19.91±8.21) than in survivors (SOFA: 3.14±2.02; APACHE II: 12.45±5.76) (p<0.001). The ROC curve analysis showed that lactate had an area under the curve (AUC) of 0.909, SOFA had an AUC of 0.809, and APACHE II had an AUC of 0.769 for predicting mortality.

Conclusions

Lactate levels are a highly sensitive predictor of mortality in critically ill patients, with significant correlations to SOFA and APACHE II scores. Lactate, as a single rapid test, provides substantial prognostic information and can aid in early triage and clinical decision-making, particularly in resource-limited settings. A single arterial lactate measurement at admission is an effective tool for predicting patient outcomes in the ICU.

## Introduction

Critical illness refers to life-threatening conditions that require mechanical or pharmacological intervention to maintain organ function. Accurate assessment of critically ill patients in the intensive care unit (ICU) is essential for treatment planning, decision-making, and family guidance [1]. While the complexity of medical interventions often defies prediction, identifying recovery patterns can assist in balancing treatment costs and benefits [2].

Since the 1980s, various models have been developed to predict illness severity and mortality in the ICU, including the Sequential Organ Failure Assessment (SOFA) and Acute Physiology and Chronic Health Evaluation (APACHE) II and III [3]. These models have facilitated demographic studies and outcome predictions, primarily focusing on mortality, organ failure, hospital stay, and quality of life [4]. Outcome prediction is vital for improving care quality and guiding resource allocation, particularly for high-cost ICU treatments and end-of-life decisions [5].

Despite these advances, research on the prognostic value of dynamic lactate variations in critically ill patients remains limited. Elevated lactate levels, particularly time-weighted averages, may predict survival beyond 30 days [6]. A multicenter study demonstrated that dynamic lactate changes correlate with improved short-term survival, especially with lactate clearance above 10% [7]. Hyperlactatemia has emerged as an independent biomarker of mortality, with higher mortality rates in patients exhibiting sustained lactate elevation over 24 hours [8]. To date, all models used to evaluate prognosis have been based on multiple parameters and tests. Therefore, this study aims to determine the prognostic value of blood lactate levels at the time of admission in critically ill patients presenting to the emergency department and their correlation with patient outcomes.

## Materials and methods

**Figure 1:**
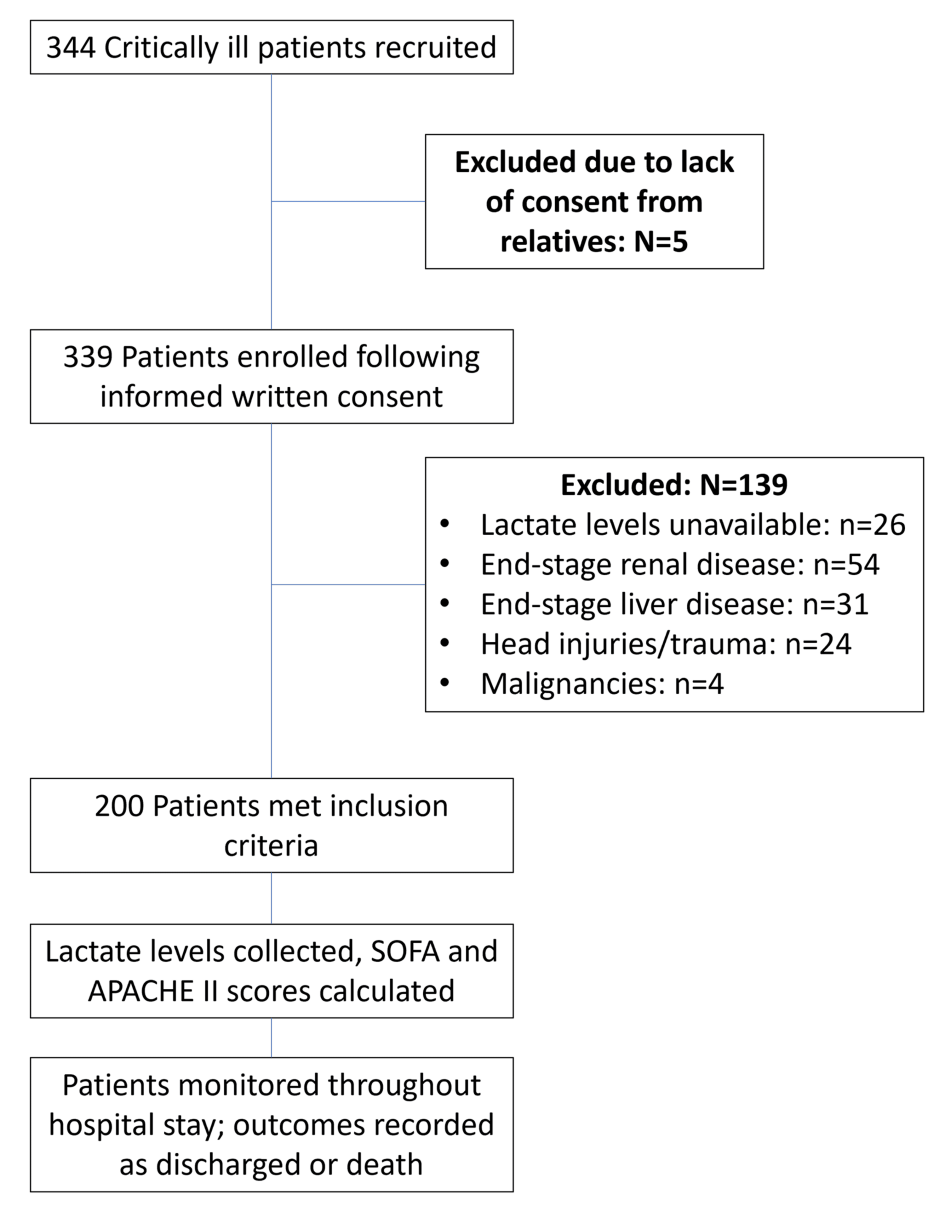
Patient selection flowchart for inclusion. SOFA: Sequential Organ Failure Assessment; APACHE II: Acute Physiology and Chronic Health Evaluation II

This descriptive cross-sectional study included 200 patients from the emergency department over one year (May 2023 to April 2024). The sample size was 200 patients. Participants were selected based on inclusion criteria: patients aged 18 years or older, hospitalized for any critical illness, and consenting to participate. Exclusion criteria included patients with end-stage renal disease (ESRD) on maintenance hemodialysis, end-stage liver disease (ESLD), malignancy, or admission due to surgical illness, head injury, or polytrauma. Figure 1 presents a flowchart for the patient selection criteria. The Institutional Ethics Committee of Maharishi Markandeshwar Institute of Medical Sciences and Research approved the study design (approval number: IEC/2518/2023).

All selected patients were informed about the nature of the study, and written informed consent was obtained in a language they understood. Data were collected on demographics, clinical findings, laboratory results, comorbidities, and inotrope support requirements. Upon admission, patients underwent routine biochemical evaluations, including complete blood count, liver function tests, renal function tests, arterial blood gas with lactate levels, random blood glucose, electrocardiography, and other investigations (blood culture, urine culture, and two-dimensional echocardiography). As per protocol, blood samples for lactate level analysis (0.5-1 mL) were collected within 30 minutes of arrival at the emergency department. Blood lactate levels were measured immediately using the ABL 800 FLEX blood gas analyzer (Radiometer Medical ApS, Brønshøj, Denmark).

The SOFA and APACHE II scores were calculated for each patient at the time of admission. Patients were monitored throughout their hospital stay, and prognosis was determined based on discharge or mortality. According to the Sepsis-3 criteria, sepsis was defined as life-threatening organ dysfunction caused by a dysregulated host response to infection. Organ dysfunction was identified as an acute change in total SOFA score of ≥2 points due to infection [9].

Statistical analysis

Statistical analysis was performed using IBM SPSS Statistics for Windows, Version 27.0 (Released 2020; IBM Corp., Armonk, New York, United States). Quantitative variables were summarized using measures of central tendency. An independent t-test was used to compare the means of normally distributed quantitative variables, and the chi-squared test was used to compare two qualitative groups. All tests were conducted with a 95% confidence interval (CI) and a significance level of 5% after dividing the study population into survival and non-survival groups. Receiver operating characteristic (ROC) curve analysis was performed to identify significant differences in the area under the curve (AUC) for lactate levels, SOFA score, and APACHE II score in predicting mortality. Sensitivity and specificity were calculated using diagnostic testing.

## Results

The mean age of the 200 study subjects was 56.8±16.9 years. Of the participants, 110 (55%) were men, and 90 (45%) were women. A total of 106 (53%) subjects were admitted for infectious causes, and 94 (47%) were admitted for non-infectious causes, as shown in Table 1. Among the 106 infectious cases, 94 (47%) met the criteria for sepsis, while 106 (53%) comprised the non-sepsis group.

**Table 1:** Underlying etiology for the admission of the study subjects. COPD: chronic obstructive pulmonary disease

System involved		N (%)
Infection (n=106)	Pulmonary infections	62 (31%)
	Gastrointestinal system infections	12 (6%)
	Central nervous system infections	7 (3.5%)
	Renal+genitourinary infections	17 (8.5%)
	Other infections	8 (4%)
Pulmonary system (COPD, bronchial asthma)		13 (6.5%)
Central nervous system (stroke, seizures)		23 (11.5%)
Cardiovascular system (heart failure)		38 (19%)
Gastrointestinal system (pancreatitis, hepatitis)		4 (2%)
Poisoning		11 (5.5%)
Others		5 (2.5%)
Total		200 (100%)

Overall, 79 subjects (39.5%) were non-survivors, while 121 (60.5%) were survivors. As presented in Table 2, the mean Glasgow Coma Scale (GCS) and mean arterial pressure (MAP) were significantly lower in the non-survivor group (GCS: 10.85±3.76; MAP: 81.38±22.44 mmHg) compared to the survivor group (GCS: 14.20±1.74; MAP: 89.63±19.14 mmHg), with p-values of <0.001 and <0.002, respectively. Additionally, the mean total leukocyte count (TLC), serum creatinine, fraction of inspired oxygen (FiO2), and lactate levels were significantly higher in the non-survivor group (TLC: 15.62±9.41×10³/μL; serum creatinine: 1.84±1.59 mg/dL; FiO2: 49.25±28.62%; lactate: 3.56±1.90 mmol/L) compared to the survivor group, with p-values of <0.011, <0.013, <0.001, and <0.001, respectively.

**Table 2:** Correlation of various parameters between the survivor and non-survivor groups. *Significant at p<0.05 SD: standard deviation; IQR: interquartile range; PaO2: partial pressure of oxygen; HCO3: bicarbonate; FiO2: fraction of inspired oxygen

Parameter	Non-survivors (n=79)			Survivors (n=121)			P-value
	Mean±SD	Median	IQR	Mean±SD	Median	IQR	
Pulse (per minute)	103.06±25.47	100	35	99.88±17.64	98	24	0.338
Systolic blood pressure (mmHg)	110.90±32.62	110	40	121.52±27.78	122	29	0.005*
Diastolic blood pressure (mmHg)	66.75±18.76	70	30	73.80±15.71	70	23	0.005*
Mean arterial pressure (mmHg)	81.38±22.44	83	27	89.63±19.14	90	25	0.002*
Respiratory rate (per minute)	24.09±7.73	24	10	22.57±5.76	22	8	0.087
Temperature (Fahrenheit)	98.43±1.08	98.30	0.70	98.25±0.96	98.20	0.90	0.235
Glasgow Coma Scale	10.85±3.76	11	6	14.20±1.74	15	1	0.001*
Platelet count (1000/mm^3^)	213.14±130.03	178	161.50	222.34±124.50	201	131.50	0.502
Packed cell volume (%)	32.97±7.52	33	10.90	33.39±8.36	33	10	0.791
Total leucocyte count (1000/mm^3^)	15.62±9.41	13.90	9.10	12.59±6.64	11.40	7.07	0.011*
Creatinine (mg/dL)	1.84±1.59	1.37	1.57	1.43±0.77	1.19	0.85	0.013*
Sodium (mEq/L)	138.67±6.97	139	6	139.13±10.42	138	6	0.680
Potassium (mEq/L)	4.09±0.71	4	0.85	4.15±0.58	4.10	0.65	0.563
Total bilirubin (mg/dL)	2.26±10.19	0.69	0.68	1.99±9.47	0.60	0.63	0.809
pH	7.30±0.16	7.33	0.21	7.37±0.09	7.40	0.08	0.001*
PaO2 (mmHg)	89.65±52.22	75	72	84.72±37.78	83.30	25.75	0.439
HCO3 (mmol/L)	21.47±7.66	21	7.20	22.79±5.53	22.30	6.45	0.076
FiO2 (%)	49.25±28.62	40	40	28.76±16.76	21	8.50	0.001*
Lactate (mmol/L)	3.56±1.90	2.80	2.80	1.47±0.82	12	0.70	0.001*

As shown in Table 3, among the 106 non-sepsis subjects, 77 (72.6%) were survivors, and 29 (27.3%) were non-survivors. The mean lactate levels were 1.33±0.90 mmol/L in survivors and 3.41±1.97 mmol/L in non-survivors. Among the 94 sepsis subjects, 44 (46.8%) were survivors, and 50 (53.1%) were non-survivors, with mean lactate levels of 1.57±0.64 mmol/L in survivors and 3.65±1.88 mmol/L in non-survivors. Lactate levels were significantly higher in non-survivors in both sepsis and non-sepsis groups.

**Table 3:** Comparison of lactate levels between non-sepsis and sepsis group of patients. *Significant at p<0.05 SD: standard deviation

Type	Non-sepsis group		Sepsis group		P-value
	Number of subjects (%)	Mean lactate levels±SD	Number of subjects (%)	Mean lactate levels±SD	
Survivors	77 (72.6%)	1.33±0.90	44 (46.8%)	1.57±0.64	0.001*
Non-survivors	29 (27.3%)	3.41±1.97	50 (53.1%)	3.65±1.88	
Total	106 (100%)	1.9±1.57	94 (100%)	2.6±1.77	

**Figure 2:**
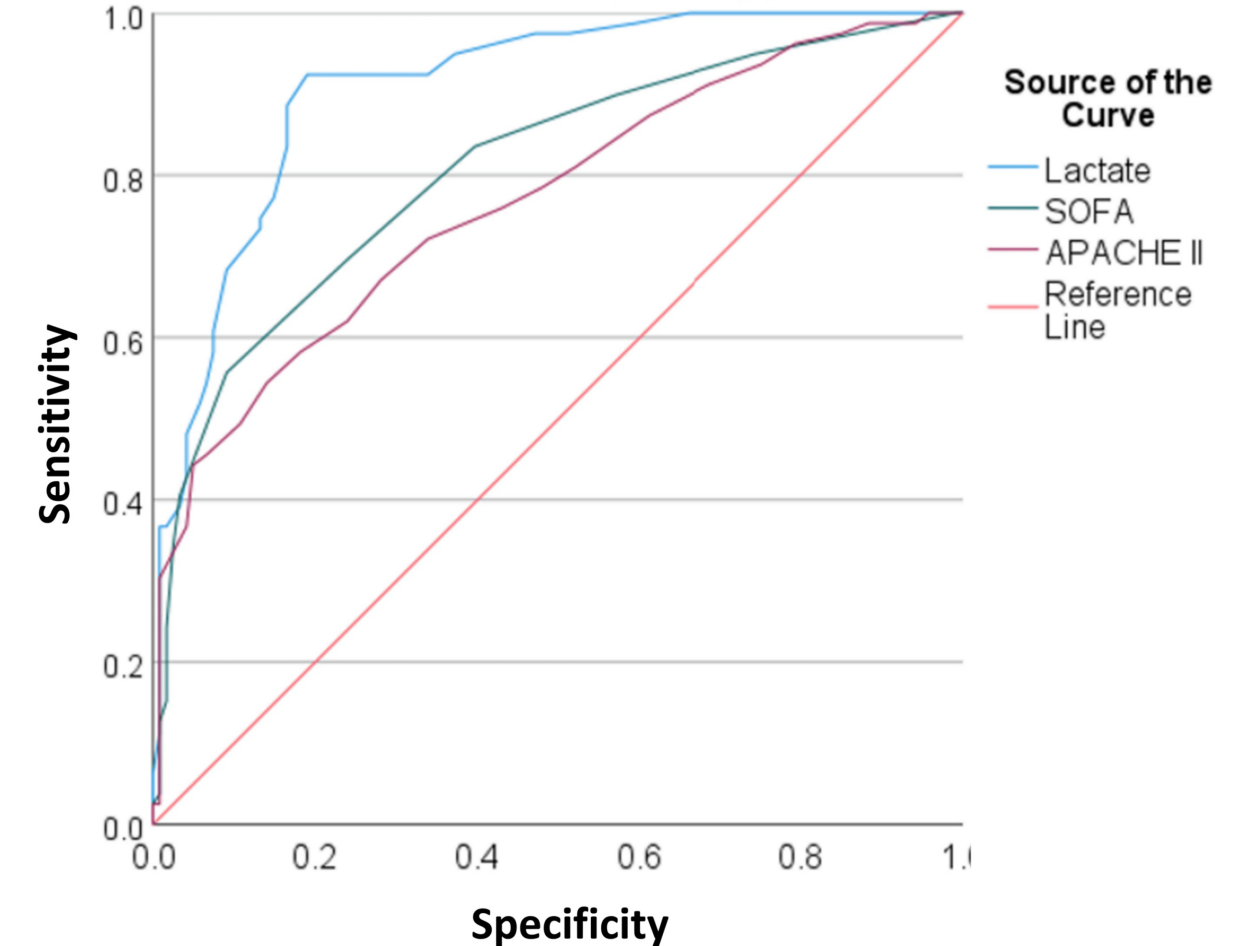
ROC of lactate, SOFA score, and APACHE II score. ROC: receiver operator characteristics; SOFA: Sequential Organ Failure Assessment; APACHE II: Acute Physiology and Chronic Health Evaluation II

Table 4 demonstrates that the mean lactate level was significantly higher in non-survivors (3.56±1.90 mmol/L) compared to survivors (1.47±0.82 mmol/L; p<0.001). Similarly, the mean SOFA score was significantly higher in non-survivors (6.35±3.19) compared to survivors (3.14±2.02; p<0.001), and the mean APACHE II score was significantly higher in non-survivors (19.91±8.21) compared to survivors (12.45±5.76; p<0.001). Figure 2 illustrates the ROC curves for lactate, SOFA, and APACHE II scores.

**Table 4:** Predictors of mortality in critical care. *Significant at p<0.05 SD: standard deviation; IQR: interquartile range; SOFA: Sequential Organ Failure Assessment; APACHE II: Acute Physiology and Chronic Health Evaluation II

Mortality predictor	Non-survivors			Survivors			P-value
	Mean±SD	Median	IQR	Mean±SD	Median	IQR	
Lactate	3.56±1.90	2.80	2.80	1.47±0.82	1.30	0.70	0.001*
SOFA	6.35±3.19	6	4	3.14±2.02	3	3	0.001*
APACHE II	19.91±8.21	19	12	12.45±5.76	12	7.50	0.001*

Table 5 presents the predictive models for mortality for lactate, SOFA, and APACHE II scores. The cut-off values for lactate, SOFA, and APACHE II scores were ≥2.25 mmol/L, ≥4.50, and ≥19.50, respectively. The sensitivity and specificity for lactate were 73% and 87%, for SOFA were 69% and 76%, and for APACHE II were 49% and 90%, respectively. The AUC was statistically significant for all three parameters: lactate (0.909, 95% CI: 0.869-0.949), SOFA (0.809, 95% CI: 0.746-0.872), and APACHE II (0.769, 95% CI: 0.701-0.837).

**Table 5:** Comparison of AUROC analysis for mortality between lactate, SOFA, and APACHE II. AUROC: area under the receiver operating characteristic; SOFA: Sequential Organ Failure Assessment; APACHE II: Acute Physiology and Chronic Health Evaluation II

Scores	Cut-off	Sensitivity	Specificity	Area	Standard error
Lactate	2.25	73%	87%	0.909	0.020
SOFA	4.50	69%	76%	0.809	0.032
APACHE II	19.50	49%	90%	0.769	0.035

## Discussion

This study evaluated the prognostic value of serum lactate levels in critically ill patients and compared their utility with established tools such as the SOFA and APACHE II scores. Consistent with prior research, our findings confirm the strong prognostic utility of lactate levels, particularly for rapid assessment in resource-limited settings [8-15].

Most patients in our study were admitted with infections, most commonly pulmonary infections, similar to findings by Atik et al. and Ma et al. [8,10]. Our observed mortality rate of 39.5% aligns with previous reports from Marty et al., Yu et al., and Tejaswini et al., who found mortality rates between 30.5% and 44.6% [11-13]. Non-survivors in our study had significantly lower GCS and MAP scores, as well as higher TLC, serum creatinine, and FiO₂, supporting the results of Tejaswini et al., Erdoğan and Findikli, and Jaiswal et al. [13-15].

Our results demonstrate that lactate levels were significantly higher in non-survivors, in both sepsis and non-sepsis cases. The area under the receiver operating characteristic (AUROC) curve for lactate was 0.909, with a sensitivity of 73%, higher than SOFA (69%) and APACHE II (49%). These findings are consistent with studies by Ma et al. and Juneja et al., which showed similar AUROC values and sensitivity for lactate as a predictor of mortality [10,16]. APACHE II, being multifactorial, showed greater specificity (90%), yet lactate, as a single test, offers practical advantages for rapid triage in critically ill patients upon admission. Cao et al., however, found higher lactate and APACHE II scores in their non-survivor group, which contrasts with our results [17].

Lactate levels also showed significant positive correlations with both the SOFA and APACHE II scores (Pearson correlation: 0.544 and 0.498, respectively), similar to findings reported by Atik et al. [8]. This supports the idea that lactate can complement these established scoring systems in clinical decision-making. As a single biomarker, lactate is a useful tool for risk stratification, guiding the need for closer monitoring and aggressive interventions.

Our study had several important limitations. The study's sample size was limited, and patients were enrolled from a single rural institution in India with a relatively resource-limited setting. Standards of care may differ from those in more advanced, technologically equipped ICUs. Patients who left against medical advice were not followed up, which may have contributed to our population's lower mean serum lactate levels. Due to financial constraints, only a single lactate reading was taken. Additionally, patients with preexisting ESLD, ESRD on maintenance hemodialysis, and malignancy and those admitted for surgical illness, head injury, or polytrauma were excluded from the study.

## Conclusions

Lactate levels are more sensitive than the SOFA and APACHE II scores in predicting mortality, though they are slightly less specific than APACHE II. In our study, elevated TLC, serum creatinine, FiO₂ requirement, and lower MAP and GCS scores were independent predictors of mortality. Our findings suggest that lactate levels, SOFA score, and APACHE II score demonstrate a strong discriminatory ability in predicting mortality among critically ill patients, with significant correlations between these parameters. Specifically, lactate levels at admission were highly sensitive in predicting patient outcomes, indicating that early lactate assessment can aid in triaging critically ill patients and inform clinical decision-making, particularly in resource-limited settings. A single arterial lactate measurement at the time of admission provides substantial prognostic information in critically ill patients.
